# Supplementary material for: Calcineurin Gamma Catalytic Subunit PPP3CC Inhibition by miR-200c-3p Affects Apoptosis in Epithelial Ovarian Cancer
Source: Genes (Basel). 2021 Sep 10;12(9):1400. doi: 10.3390/genes12091400 (PMC8470066; doi:10.3390/genes12091400)
Supplement: Supplementary file 1 [file genes-12-01400-s001.zip › Supplementary Figure S1_S2_S3.pdf]

Supplementary Figures

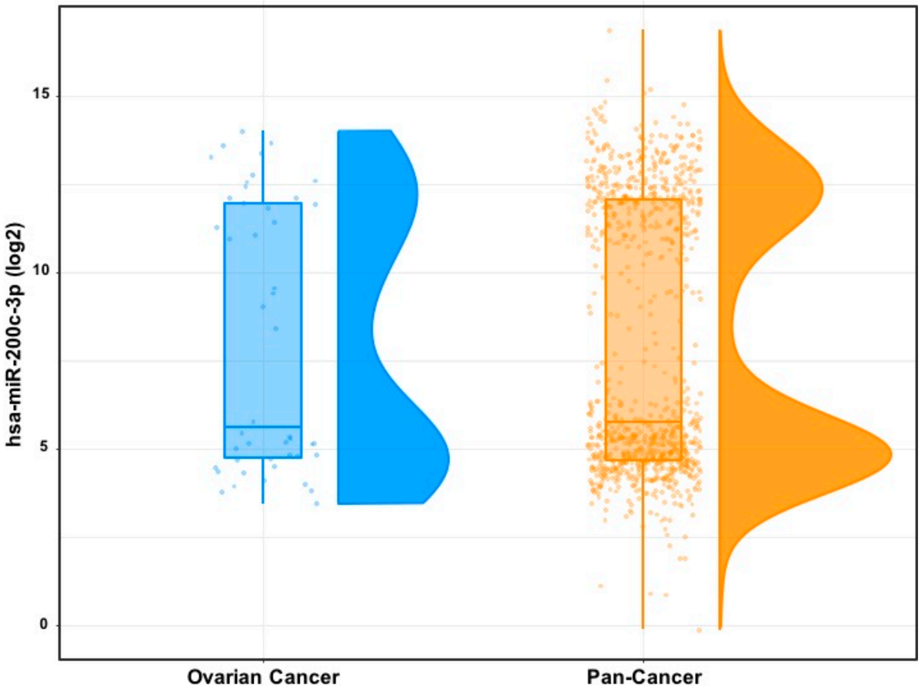

Figure S1: Raincloud plot showing the bimodal distribution of miR-200c-3p expression both in 46 OC cell lines and at pan-cancer level (988 cell lines across 24 different primary sites) by evaluating CCLE data.

Supplementary Figures

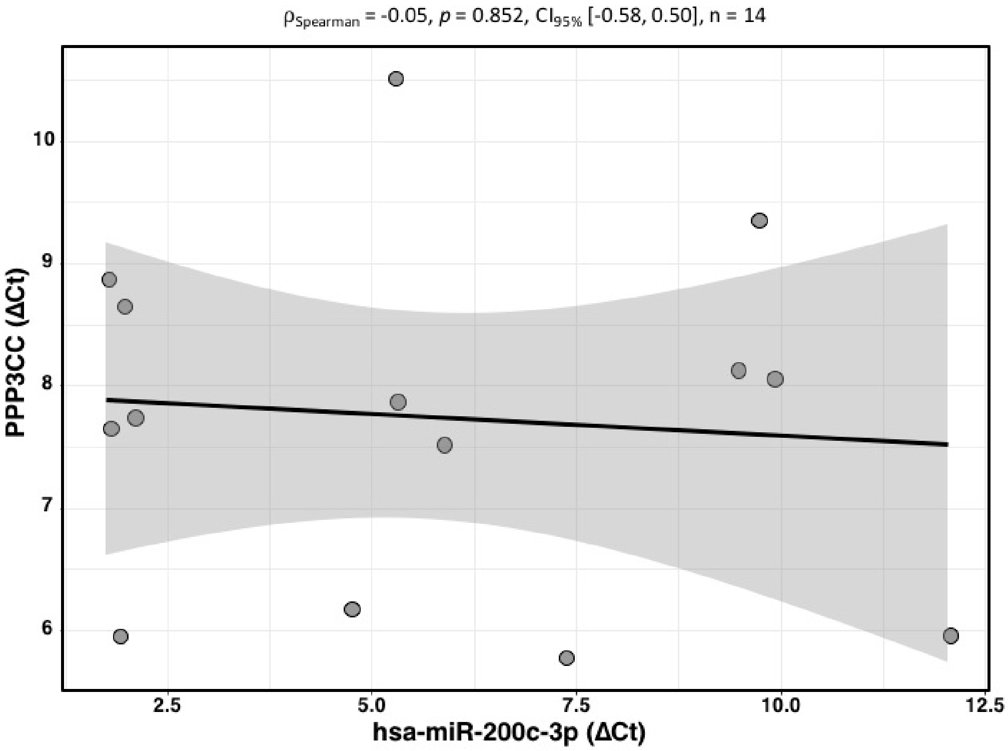

Figure S2: Anticorrelation analysis of miR-200c-3p and PPP3CC in the 14 controls. Spearman's rank correlation coefficient was equal to -0.05 with p-value 0.852.

Supplementary Figures

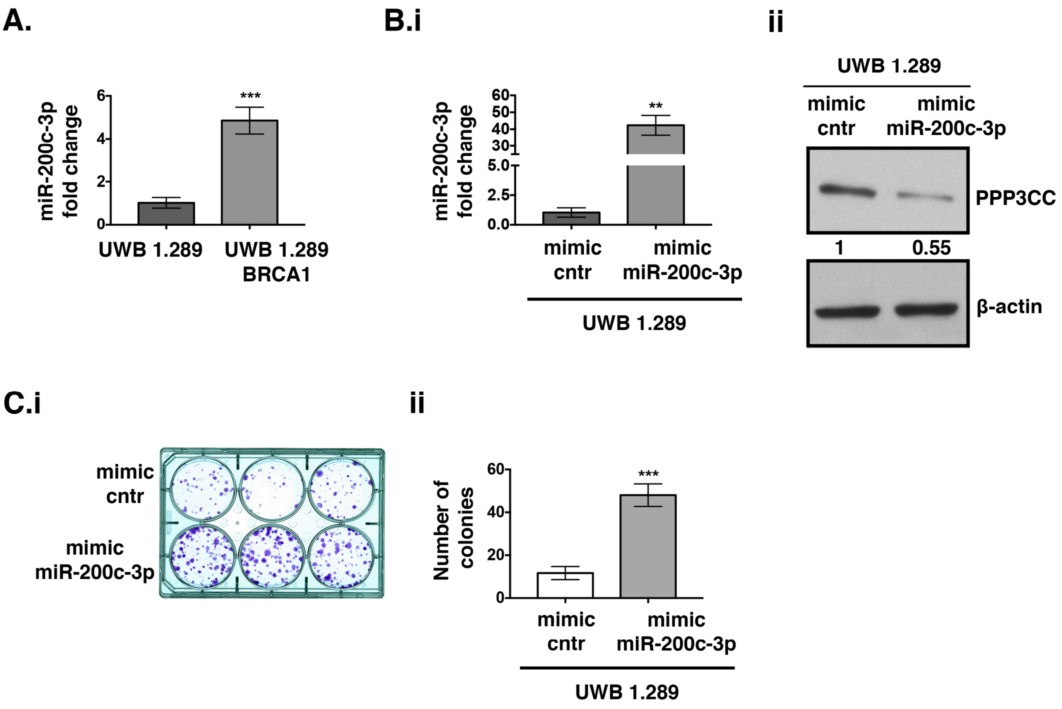

Figure S3

Figure S3: miR-200c-3p over-expression in UWB 1.289 cells decreases PPP3CC expression and increases clonogenic capacity. A: detection of miR-200c-3p in UWB 1.289 and UWB 1.289+BRCA1 was performed by RT-qPCR. RNU6 was used as a reference gene. (Bi): miR-200c-3p mimics were transfected in UWB 1.289 for 72h. MiR-200c-3p expression was assessed by RT-qPCR. RNU6 was used as a reference gene. Fold change expression was calculated in terms of $2^{-\Delta\Delta Ct}$. (Bii): Western blot (WB) analysis of PPP3CC expression. β-actin was used as a loading control. Values show the fold change of PPP3CC expression in mimic-miR-200c-3p transfected cell line comparing to the mimic control (cntr), by Image J densitometric analysis. (Ci): 72h post-transfected cells ($2x10^3$) were seeded in a 6-well plate and stained with crystal violet after 10 days to estimate colony formation ability (Cii) the average number of colonies in each well of the clonogenic assays were measured with the count tool of PhotoshopCC2017. The experiments were performed in triplicates and were repeated at least twice. Two-tailed unpaired t-test was applied for statistical significance, **p<0.01, ***p<0.001.
