# Supplementary material for: Calcineurin Gamma Catalytic Subunit PPP3CC Inhibition by miR-200c-3p Affects Apoptosis in Epithelial Ovarian Cancer
Source: Genes (Basel). 2021 Sep 10;12(9):1400. doi: 10.3390/genes12091400 (PMC8470066; doi:10.3390/genes12091400)
Supplement: Supplementary file 1 [file genes-12-01400-s001.zip › Supplementary materials and methods.pdf]

Supplementary materials and methods

Transfection with mimic-miR-200c-3p

Exponentially growing UWB1.289 cells (ATCC CRL-2945; Manassas, VA, USA) were plated in a six well plate at $0.3x10^6$ cells per well, to reach 70-80% confluency. The cells were transfected with 40 nM mimic miR-200c-3p oligonucleotide (MISSION®, Sigma-Aldrich; Cat. n.HMI0354-5NMOL), using DharmaFECT Duo Transfection Reagent (Dharmacon; Cat. n. T-2010-02), according to the manufacturer's instructions. The cells were harvested at 72h post transfection for total RNA and protein extraction. RT-qPCR, western blots and clonogenic assay were performed as described in the main text, materials and methods section .
