# Supplementary material for: Calcineurin Gamma Catalytic Subunit PPP3CC Inhibition by miR-200c-3p Affects Apoptosis in Epithelial Ovarian Cancer
Source: Genes (Basel). 2021 Sep 10;12(9):1400. doi: 10.3390/genes12091400 (PMC8470066; doi:10.3390/genes12091400)
Supplement: Supplementary file 1 [file genes-12-01400-s001.zip › Supplementary tables S1 and S2.pdf]

Supplementary Tables.

Table S1: List of the 11 genes selected as potential miR-200c-3p target genes through the anti-correlation estimated from CCLE and TCGA datasets

Gene name	Correlation_CCLE * (ρ)	Correlation_TCGA* (ρ)	Tumor/Control_TCGA@ (fold change)
ZEB1	-0.723448872721684	-0.249055204950147	0.451243596169514
CRTAP	-0.526981190255936	-0.135253837528752	0.709721570516992
PMP22	-0.587437170713167	-0.284079942031707	0.500270380554825
PPP3CC	-0.473697193956213	-0.140368482737894	0.715175342230939
SAP30	-0.543509096515572	-0.233056808485156	0.866972274672618
SLC35B4	-0.454422105218531	-0.171975345071155	0.826747458105071
ST3GAL2	-0.521956335025031	-0.124821288012663	0.796238888540155
SYDE1	-0.664158623770088	-0.1652523847293	0.651303399918923
ZEB2	-0.396237475798043	-0.148200787981102	0.644807180881513
KDELC1	-0.513429338182374	-0.295403588202153	1.56545108565678
ZCCHC24	-0.530558125192723	-0.306491963265963	0.342374163192311

*Spearman Correlation coefficients (ϱ) reported in the second and third columns, @TargetScan predictions and differential expression between tumor and normal ovarian tissue samples from TCGA (fourth column: fold expression in tumors compared to normal tissues).

Table S2: Enrichment results applied to the list of the 11 genes selected as potential miR-200c-3p target genes using ConsensusPathDB annotations.

Pathway*	Source	Genes
Hypoxia-mediated EMT and Stemness	Wikipathways	ZEB1
TGF-beta Signaling Pathway	Wikipathways	ZEB1;ZEB2
Epithelial to mesenchymal transition in colorectal cancer	Wikipathways	ZEB1;ZEB2
sumoylation as a mechanism to modulate ctbp-dependent gene responses	BioCarta	ZEB1
Type I collagen synthesis in the context of Osteogenesis imperfecta	Wikipathways	CRTAP
Extracellular matrix organization	Reactome	CRTAP
Collagen biosynthesis and modifying enzymes	Reactome	CRTAP
Collagen formation	Reactome	CRTAP
Neural Crest Differentiation	Wikipathways	PMP22

Nervous system development	Reactome	PMP22
Developmental Biology	Reactome	PMP22
EGR2 and SOX10-mediated initiation of Schwann cell myelination	Reactome	PMP22
Activation of BH3-only proteins	Reactome	PPP3CC
**R**ole of mef2d in t-cell apoptosis	BioCarta	PPP3CC
Intrinsic Pathway for Apoptosis	Reactome	PPP3CC
Programmed Cell Death	Reactome	PPP3CC
Epigenetic regulation of gene expression	Reactome	SAP30
Epstein-Barr virus infection - Homo sapiens (human)	KEGG	SAP30
Chromatin modifying enzymes	Reactome	SAP30
Chromatin organization	Reactome	SAP30
Proteoglycan biosynthesis	EHMN	SLC35B4
Galactose metabolism	EHMN	SLC35B4
Transport of vitamins, nucleosides, and related molecules	EHMN	SLC35B4
SLC-mediated transmembrane transport	Reactome	SLC35B4
Asparagine N-linked glycosylation	Reactome	ST3GAL2
Metabolism of carbohydrates	Reactome	ST3GAL2
Post-translational protein modification	Reactome	ST3GAL2
Metabolism of proteins	Reactome	ST3GAL2
RHOF GTPase cycle	Reactome	SYDE1
RHOQ GTPase cycle	Reactome	SYDE1
RAC2 GTPase cycle	Reactome	SYDE1

CDC42 GTPase cycle	Reactome	SYDE1
Mammary gland development pathway - Embryonic development (Stage 1 of 4)	Wikipathways	ZEB2
TGF-beta Receptor Signaling	Wikipathways	ZEB2
TGF-beta receptor signaling in skeletal dysplasias	Wikipathways	ZEB2
MicroRNAs in cancer - Homo sapiens (human)	KEGG	ZEB1;ZEB2

*The total number of annotated pathways were 153. Four annotated pathways were enlisted for each gene.
